# *Notes from the Field:* Severe *Vibrio vulnificus* Infections During Heat Waves — Three Eastern U.S. States, July–August 2023

**DOI:** 10.15585/mmwr.mm7304a3

**Published:** 2024-02-01

**Authors:** Michael J. Hughes, Eileen Flaherty, Nicole Lee, Amy Robbins, Daniel L. Weller

**Affiliations:** ^1^Division of Foodborne, Waterborne, and Environmental Diseases, National Center for Emerging and Zoonotic Infectious Diseases, CDC; ^2^Connecticut Department of Public Health; ^3^North Carolina Department of Health and Human Services; ^4^New York State Department of Health.

**Summary**

**What is already known about this topic?**

*Vibrio vulnificus*, a waterborne and foodborne pathogen, can infect open wounds through contact with salt water, brackish water, or raw seafood. Infections can also occur after consuming raw or undercooked seafood.

**What is added by this report?**

During July–August 2023, 11 cases of severe *V. vulnificus* infection were reported among residents of three eastern U.S. states after a period of heat waves and elevated sea surface temperatures. Patients reported multiple routes of exposure; four patients experienced septic shock; five patients died.

**What are the implications for public health practice?**

Avoiding open wound contact with brackish water, salt water, and raw seafood; and thoroughly cooking oysters and other seafood before eating can prevent infection and illness.

*Vibrio vulnificus*, a waterborne and foodborne pathogen, lives in estuarine environments and thrives in warmer waters. *V. vulnificus* can infect open wounds through contact with salt water, brackish water, or raw seafood; infections can also occur after consuming raw or undercooked seafood. In the United States, 150–200 *V. vulnificus* infections are reported to CDC annually, approximately 20% of which are fatal.* During June–August 2023, widespread heat waves and above-average sea surface temperatures occurred in the United States (*1*). During July–August 2023, public health officials in three eastern U.S. states (Connecticut, New York, and North Carolina) were notified of *V. vulnificus* infections associated with exposure to coastal waters and seafood, most of which were severe and led to septic shock or death. This report describes *V. vulnificus* infections among residents of these three states during the 2023 heat waves.

## Investigation and Outcomes

After being notified of positive *Vibrio* clinical test results through routine disease surveillance, public health officials in all states interview patients using the Cholera and Other *Vibrio* Illness Surveillance case report form,^†^ which collects information on underlying conditions, clinical outcomes, and exposures occurring before illness onset. For cases involving raw oyster consumption, investigators contact retail facilities, collect oyster harvest tags, and notify relevant state and federal authorities after reports of possibly contaminated raw shellfish shipped from out of state. This activity was reviewed by CDC, deemed not research, and was conducted consistent with applicable federal law and CDC policy.^§^

### Characteristics of Cases

During July–August 2023, 11 persons infected with *V. vulnificus* were reported to health officials in North Carolina (seven), Connecticut (two), and New York (two) (Figure). The median patient age was 70 years (range = 37–84 years). Seven patients were male. One North Carolina patient was lost to follow-up. Among 10 patients with information available, all but one had at least one underlying condition, most commonly diabetes (three), cancer (three), heart disease (three), history of alcoholism (three), and hematologic disease (two). Six patients experienced either septic shock (four) or died (five); three experienced both. All of the patients who died had at least one underlying condition.

**FIGURE:**
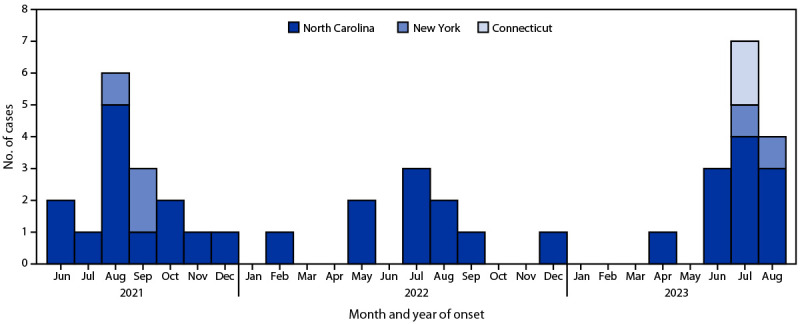
Number of *Vibrio vulnificus* infections, by illness onset date and patient state of residence (N = 41) — Connecticut, New York, and North Carolina, June 2021–August 2023

### Likely Routes of *V. vulnificus* Exposure

Waterborne transmission of *V. vulnificus* resulting from wound exposure to marine or estuarine water along the U.S. Atlantic coast during July 7–August 22, 2023 was the most likely route of infection in six cases. Waterborne cases occurred among residents of North Carolina (three), New York (two), and Connecticut (one). Two additional cases in North Carolina residents likely resulted from exposure to a cut on the hand while handling raw seafood during food preparation. Among the two remaining cases with exposure information, one resulted from a foodborne exposure of a Connecticut resident who reported consuming raw oysters in another state and did not report any relevant water or environmental exposures and the second patient, a North Carolina resident, reported both wound exposure to brackish water and raw oyster consumption.

## Preliminary Conclusions and Actions

Connecticut, New York, and North Carolina public health officials rapidly identified cases of *V. vulnificus* infection and initiated investigations. All three state health departments issued press releases informing the public about *V. vulnificus* infections, and CDC issued a Health Advisory Notice.^¶^ A notable feature of these cases, beyond their severe clinical outcomes, is that they occurred in the wake of record-breaking U.S. heat waves (*2*). Although these cases reported during July–August cannot be solely attributed to the heat waves, the relationship between vibriosis incidence and environmental conditions favorable to *Vibrio* growth, namely elevated water surface temperatures and low salinity, is well-documented (*3*,*4*). Whereas North Carolina reported 10–13 cases per year during 2021–2023, Connecticut reported no *V. vulnificus* infections during all of 2021–2022, and New York reported three cases in 2021 and none in 2022 (Figure). As coastal water temperatures increase, *V. vulnificus* infections are expected to become more common (*5*). Persons can take steps to prevent illness by avoiding wound contact with brackish water, salt water, and raw seafood, and by thoroughly cooking oysters and other seafood before eating.

## Footnotes

*
https://www.cdc.gov/vibrio/wounds.html

† https://www.cdc.gov/vibrio/surveillance.html

§ 45 C.F.R. part 46.102(l)(2), 21 C.F.R. part 56; 42 U.S.C. Sect. 241(d); 5 U.S.C. Sect. 552a; 44 U.S.C. Sect. 3501 et seq.

¶
https://emergency.cdc.gov/han/2023/han00497.asp

## References

[1] National Oceanic and Atmospheric Administration. U.S. saw its 9th-warmest August on record. Washington, DC: US Department of Commerce, National Oceanic and Atmospheric Administration; 2023. https://www.noaa.gov/news/us-saw-its-9th-warmest-august-on-record

[2] National Oceanic and Atmospheric Administration. 2023 was the world’s warmest year on record, by far. Washington, DC: US Department of Commerce, National Oceanic and Atmospheric Administration; 2023. https://www.noaa.gov/news/2023-was-worlds-warmest-year-on-record-by-far

[3] Galanis E, Otterstatter M, Taylor M. Measuring the impact of sea surface temperature on the human incidence of *Vibrio* sp. infection in British Columbia, Canada, 1992–2017. Environ Health 2020;19:58. 10.1186/s12940-020-00605-x 32460848 PMC7251872

[4] Froelich BA, Daines DA. In hot water: effects of climate change on *Vibrio*-human interactions. Environ Microbiol 2020;22:4101–11. 10.1111/1462-2920.14967 32114705

[5] Archer EJ, Baker-Austin C, Osborn TJ, Climate warming and increasing *Vibrio vulnificus* infections in North America. Sci Rep 2023;13:3893. 10.1038/s41598-023-28247-2 36959189 PMC10036314

